# Microcatheter-assisted guidewire manipulation via peroral pancreatoscopy for recanalization of pancreaticojejunostomy obstruction

**DOI:** 10.1055/a-2550-4146

**Published:** 2025-03-20

**Authors:** Michihiro Yoshida, Yasuki Hori, Akihisa Kato, Hidenori Sahashi, Tadashi Toyohara, Yusuke Kito, Hiromi Kataoka

**Affiliations:** 1 Department of Gastroenterology and Metabolism, Nagoya City University Graduate School of Medical Sciences, Nagoya, Japan

Pancreatojejunostomy stricture is one of the complications after pancreaticoduodenectomy 1 . Endoscopic ultrasound-guided pancreatic duct drainage (EUS-PD) has emerged as an alternative to enteroscopy-guided treatment. We present a case demonstrating the successful coordinated manipulation of a 3-Fr microcatheter and pancreatoscopy for recanalization of a complete pancreaticojejunostomy obstruction.

A 72-year-old man, who had previously undergone pancreaticoduodenectomy for distal bile duct cancer, presented with pancreatitis due to a pancreaticojejunostomy anastomotic stricture ( Fig. 1 **a** ). As the anastomosis could not be identified by the enteroscopy-guided approach, EUS-PD was performed. The anastomosis was completely stenosed on contrast imaging and an attempt to negotiate the obstruction using a guidewire under fluoroscopic guidance failed; therefore, a plastic stent was deployed in the main pancreatic duct ( Fig. 1 **b** ).

**Fig. 1:**
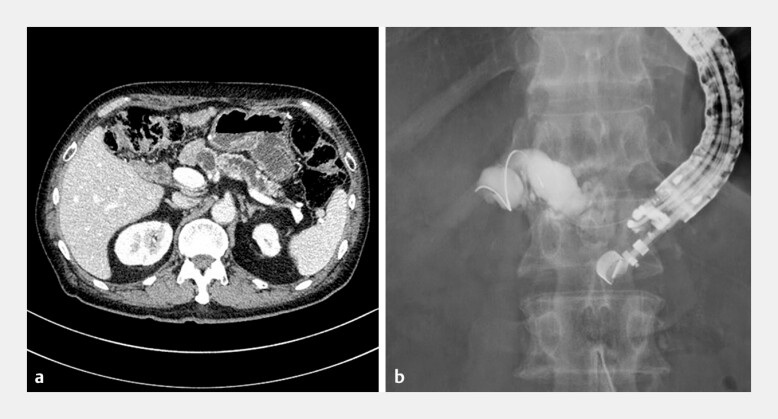
Initial imaging. **a** Computed tomography scan showed dilation of the main pancreatic duct. **b** Attempts to advance the guidewire through the obstruction were unsuccessful.

Another attempt to advance the guidewire through the anastomotic obstruction was unsuccessful 3 months after EUS-PD. A covered metal stent (Hanaro Benefit; Boston Scientific, Marlborough, Massachusetts, USA) was placed over the fistula of the pancreaticogastrostomy. Then, 1 week later, the SpyGlass DS digital single-operator cholangioscope (Boston Scientific) was inserted into the pancreatic duct via the metal stent. However, the obstruction could still not be breached with guidewire manipulation under peroral pancreatoscopy. A 3-Fr microcatheter (Daimon-ERCP-catheter; Hanaco Medical, Saitama, Japan) was then introduced through the pancreatoscope, and guidewire manipulation under the microcatheter enhanced maneuverability, facilitating breakthrough and enabling successful penetration of the anastomotic site ( Fig. 2 ). After confirming the jejunum using contrast injection through the microcatheter, a plastic stent was successfully placed transgastrically to bridge the pancreatic duct and jejunal anastomosis in an antegrade fashion ( Video 1 ).

**Fig. 2:**
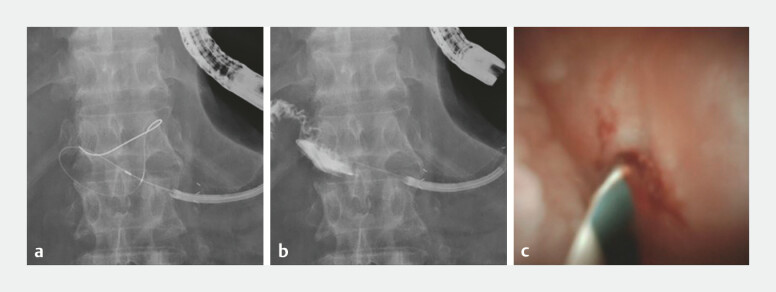
Successful guidewire placement. **a** Coordinated manipulation of the guidewire and a 3-Fr microcatheter (Daimon-ERCP-catheter; Hanaco Medical, Saitama, Japan) under the SpyGlass DS digital single-operator cholangioscope (Boston Scientific, Marlborough, Massachusetts, USA) allowed successful penetration of the anastomotic site. **b** Confirmation of the jejunum using contrast injection through the microcatheter. **c** The peroral pancreatoscopy image showed the guidewire penetrating the anastomotic site.

3-Fr microcatheter-assisted peroral pancreatoscopy via pancreaticogastrostomy for recanalization of pancreaticojejunostomy obstruction.Video 1

Peroral pancreatoscopy through the pancreaticogastrostomy fistula has been reported as an effective and safe option for antegrade guidewire placement 2 , but challenges persist, particularly when navigating the guidewire through the pancreatoscope. The microcatheter offers distinct advantages, including the ability to obtain selective contrast-enhanced images and enhanced maneuverability of the guidewire for a super-selective approach 3 .

Endoscopy_UCTN_Code_TTT_1AS_2AD

## Notes

Endoscopy E-Videos https://eref.thieme.de/e-videos
*E-Videos* is an open access online section of the journal *Endoscopy* , reporting on interesting cases and new techniques in gastroenterological endoscopy. All papers include a high-quality video and are published with a Creative Commons CC-BY license. Endoscopy E-Videos qualify for HINARI discounts and waivers and eligibility is automatically checked during the submission process. We grant 100% waivers to articles whose corresponding authors are based in Group A countries and 50% waivers to those who are based in Group B countries as classified by Research4Life (see: https://www.research4life.org/access/eligibility/ ). This section has its own submission website at https://mc.manuscriptcentral.com/e-videos .
